# Endoplasmic Reticulum Stress of Oral Squamous Cell Carcinoma Induces Immunosuppression of Neutrophils

**DOI:** 10.3389/fonc.2022.818192

**Published:** 2022-03-16

**Authors:** Ching-Fang Wu, Tzu-Ting Hung, Yu-Chieh Su, Po-Jen Chen, Kuei-Hung Lai, Chih-Chun Wang

**Affiliations:** ^1^ School of Medicine, College of Medicine, I-Shou University, Kaohsiung, Taiwan; ^2^ Division of Nephrology, Department of Internal Medicine, E-Da Cancer Hospital, Kaohsiung, Taiwan; ^3^ Division of Hematology-Oncology, Department of Internal Medicine, E-Da Hospital, Kaohsiung, Taiwan; ^4^ Department of Medical Research, E-Da Hospital, Kaohsiung, Taiwan; ^5^ PhD Program in Clinical Drug, Development of Herbal Medicine, College of Pharmacy, Taipei Medical University, Taipei, Taiwan; ^6^ Department of Otolaryngology, E-Da Hospital, Kaohsiung, Taiwan

**Keywords:** endoplasmic reticulum stress, oral squamous cell carcinoma, neutrophils, lectin-like oxidized low-density lipoprotein receptor-1 (LOX-1), immunosuppression

## Abstract

The endoplasmic reticulum (ER) stress of cancer cells not only determined cancer cell fate but also indirectly triggered proinflammatory or immunosuppressive responses of macrophages. In addition, ER stressed neutrophils were known to acquire immunosuppressive activity with surface expression of lectin-like oxidized low-density lipoprotein receptor-1 (LOX-1). Since the importance of tumor ER stress and immunosuppressive neutrophils has been emphasized in head and neck cancers, we hypothesized that the ER stress of oral squamous cell carcinoma (OSCC) could transform neutrophils into LOX-1 expressing immunosuppressive phenotype. Two human OSCC cell lines, SCC25 and OML1, were treated with either vehicle or thapsigargin (THG), an ER stress inducer. These tumor conditioned media (TCM) were collected accordingly. Then human peripheral blood neutrophils from healthy donors were cultured in these TCM. The results showed that neutrophils cultured in THG-treated TCM had higher expression of LOX-1 compared with those cultured in vehicle-treated TCM. Moreover, by interleukin-2/anti-CD3/anti-CD28 activated autologous T cell proliferation assay, neutrophils conditioned by THG-treated TCM were shown to inhibit T cell proliferation more significantly than those conditioned by vehicle-treated TCM. These novel findings indicated that the ER stress of OSCC could be transmitted to neutrophils which in turn expressed LOX-1 and obtained immunosuppressive ability. Our findings further supported the existence of “transmissible” ER stress between tumor cells and neutrophils.

## Introduction

Within tumor milieu, both cell intrinsic metabolic demands and hostile environmental conditions such as hypoxia, nutrient starvation, low pH, and free radicals can provoke endoplasmic reticulum (ER) stress and then trigger unfolded protein response (UPR) in cancer cells (1). UPR is mainly composed of three signaling pathways initiating by activating transcription factor 6 (ATF6), protein kinase R-like ER kinase (PERK) followed by eukaryotic translation initiation factor 2α (eIF2α) and activating transcription factor 4 (ATF4), or inositol-requiring enzyme 1α (IRE1α) with subsequent splicing of X-box binding protein 1 (XBP-1), together regulating the subsequent transcription of chaperons including binding immunoglobulin protein (BiP, encoded by heat shock protein family A member 5, *HSPA5*) and proteins to restore ER homeostasis and promote adaptation of cancer cells to the hostile environment. On the contrary, overwhelmed ER stress leads to cancer cell death by CCAAT/enhancer-binding protein homologous protein (CHOP)-mediated apoptosis or immunogenic cell death (1). In addition to cancer cells, ER-stressed tumor-infiltrating myeloid cells and T cells also promote an immunosuppressive microenvironment (1). Moreover, ER stress can be even transmitted from cancer cells to nearby macrophages through tumor-secreted Golgi protein (2) or certain soluble factors (3, 4), which has been termed as “transmissible” ER stress (5). These ER-stressed macrophages (2–4) then in turn acquire proinflammatory or immunosuppressive activities to facilitate tumor growth (5). 

Recently the presence of immunosuppressive neutrophils, or polymorphonuclear myeloid-derived suppressor cells (PMN-MDSCs), have been confirmed in many types of cancer (6–10). These neutrophils are known to inhibit T cell functions mainly by releasing reactive oxygen species, depleting nearby L-arginine by secretion of arginase-I, expressing programmed death ligand 1 (PD-L1) on the surface, or recruit regulatory T cells *via* secreting C-C motif chemokine ligand 17 (CCL17) (11). The identification of lectin-like oxidized low-density lipoprotein receptor-1 (LOX-1) as a marker for immunosuppressive neutrophils (8) further facilitates experimental approaches to study these neutrophils. Moreover, ER stress was found to be responsible for LOX-1 expression and corresponding immunosuppressive activities of neutrophils (8).

The importance of tumor ER stress and immunosuppressive neutrophils have been emphasized especially in head and neck cancers (HNCs). For example, tumor BiP expression were associated with higher malignant potential for precancerous lesions (12) as well as advanced cancer status and poor survival in HNCs (13, 14). Other UPR targets such as Derlin, Calnexin, and Calreticulin were also upregulated in cancer tissues of HNCs (15). On the other hand, immunosuppressive neutrophils or PMN-MDSCs, either in peripheral blood or tumor sites, were more dominant compared with non-cancer controls (16) and correlated with worse survival in HNCs (7, 8, 17). However, the possible interaction between tumor ER stress and immunosuppressive neutrophils has not yet evaluated.

Given that ER stress of cancer cells could be transmitted to nearby myeloid cells (2, 3, 18–20) and ER stress could induce immunosuppression of neutrophils (8), we hypothesized that ER stress responses in cancer cells could also manipulate neutrophils by upregulating their LOX-1 expression and immunosuppressive activity. Since tumor ER stress and immunosuppressive neutrophils both existed in HNCs and the pathogenesis of oral squamous cell carcinoma (OSCC) in Asia was different from that in Europe due to high prevalence of betel nut chewing, two human OSCC cancer cell lines derived from different races, SCC25 and OML1, were utilized in our experiments. In this *in vitro* study, the effects of conditioned medium from ER-stressed SCC25 or OML1 on neutrophils were evaluated by performing flow cytometry analysis of their LOX-1 expression as well as T cell proliferation assays to assess their immunosuppressive ability.

## Materials and Methods

### Ethics Statement

Written informed consents were obtained from all participated healthy adults and their blood was collected *via* venipuncture for the following experiments. This study was approved by Institution Review Board of E-Da Hospital (number EMRP-106-113 and EMRP-107-141) and was in adherence with the Declaration of Helsinki.

### Cell Culture and Preparation of Tumor Conditioned Medium (TCM)

SCC25, a human cancer cell line derived from squamous cell carcinoma of the tongue, was purchased from American Type Culture Collection (ATCC; Manassas, VA, USA) and maintained in DMEM/F12 (Thermo Fisher Scientific, MA, USA) medium supplemented with 2.5 mM L-glutamine (Thermo Fisher Scientific, MA, USA), 0.5 mM sodium pyruvate (Thermo Fisher Scientific, MA, USA), 10% fetal bovine serum (FBS, Hyclone Laboratories, UT, USA) and 1% penicillin/streptomycin (Thermo Fisher Scientific, MA, USA). Another domestic human oral cancer cell line OML1, originally obtained from Dr. Yong-Kie Wong (Department of Dentistry, Taichung Veterans General Hospital, Taichung, Taiwan), is a radiosensitive cell line in comparison with its offspring radioresistant OML1-R (21, 22). OML1 was maintained in RPMI 1640 medium (Thermo Fisher Scientific, MA, USA) supplemented with 2 mM L-glutamine, 10% FBS and 1% penicillin/streptomycin. TCM from SCC25 or OML1 was generated as previously reported (3) with some adaptations. RPMI 1640 medium containing 2 mM L-glutamine, 25 mM 4-(2-hydroxyethyl)-1-piperazineethanesulfonic acid (HEPES, Thermo Fisher Scientific, MA, USA), 10% FBS and 1% penicillin/streptomycin was used throughout the generation of TCM. SCC25 and OML1 cells were treated with either vehicle (0.02% DMSO) or 2 μM thapsigargin (THG) (Merck, Germany) to induce ER stress. After 4 hours of treatment, the cells were washed twice with phosphate buffered saline (PBS, Uni-Onward, Taiwan) and then re-supplemented with fresh medium for another 20 hours. Then these supernatants were collected, centrifuged at 1,500g for 10 mins, filtered, and stored at -80°C as TCM. Before use, it was returned to room temperature.

### Isolation and Culture of Human Neutrophils

Human neutrophils were isolated from the blood of healthy donors as previously described (23). In brief, ethylenediaminetetraacetic acid (EDTA)-anticoagulated blood was diluted with PBS and then underwent density gradient centrifugation at 300g, 30 mins using Ficoll-Paque™ Plus (1.077 g/ml, Merck, Germany) to obtain the neutrophil-erythrocyte layer. Then 1% polyvinyl alcohol (Merck, Germany) in 0.9% NaCl (Merck, Germany) was added at a ratio of 1:1 (v/v) to neutrophil-erythrocyte suspensions and incubated for 20-30 mins for erythrocyte sedimentation. Residual erythrocytes were further lysed with hypotonic 0.2% NaCl solution. Neutrophils isolated with this method were subsequently cultured in RPMI 1640 medium with 2 mM L-glutamine, 25 mM HEPES, 10% FBS, 1% penicillin/streptomycin or TCM, both containing 10 ng/ml granulocyte-macrophage colony-stimulating factor (GM-CSF, Peprotech, NJ, USA) to maintain viability of neutrophils. As a positive control, 0.5 μM THG was given directly to neutrophils to induce their ER stress. As for coculture of neutrophils and T cells, the dose of THG treatment on neutrophils from different donors has been titrated to obtain acceptable responses with preserved viability. Therefore, a lower dose of THG (0.1 μM) was used for neutrophils from some donors instead. During incubation, neutrophils were cultured at 37°C under a humidified atmosphere of 5% CO_2_ and 95% air.

### Flow Cytometry

Human neutrophils were first blocked with FcR blocking reagent (Miltenyi Biotech, Germany) and then stained with Fixable Viability Stain 520 (1:4000 dilution, BD Biosciences, NJ, USA) and phycoerythrin (PE)-conjugated anti-LOX-1 antibody (clone 15C4, 1:800 dilution, BioLegend, CA, USA). UltraComp eBeads Compensation Beads (Thermo Fisher Scientific, MA, USA) were used for compensation. Flow cytometry data were acquired using BD Accuri C6 Flow Cytometer (BD Biosciences, NJ, USA) and analyzed by Flowjo v10.6.2 software (BD Biosciences, NJ, USA).

### T Cell Proliferation Assay

The autologous non-specific T cell activation by interleukin-2 (IL-2)/anti-CD3/anti-CD28 system was utilized to evaluate immunosuppression of neutrophils. Human neutrophils were isolated as mentioned above. At the same time, peripheral blood mononuclear cell (PBMC) layer was also obtained from the same donor after density gradient centrifugation. Then untouched T cells were isolated from PBMCs by using EasySep Human T Cell Isolation Kit (STEMCELL Technologies, Canada) and subsequently labeled with 5 μM carboxyfluorescein succinimidyl ester (CFSE) (Abcam, United Kingdom). Finally CFSE-stained T cells (2x10^5^ cells/200ul/well) and neutrophils from the same donors were cultured in RPMI 1640 containing 150 μM L-Arginine (Merck, Germany), 55 μM 2-mercaptoethanol (Thermo Fisher Scientific, MA, USA), multi-vitamins (Hyclone Laboratories, UT, USA), non-essential amino acids (Thermo Fisher Scientific, MA, USA), 1 mM sodium pyruvate, 10% FBS, 1% penicillin/streptomycin, 0.1 μg/mL IL-2 (Peprotech, NJ, USA), and 25 ul/ml ImmunoCult CD3/CD28 T cell Activator (STEMCELL Technologies, Canada) in 96-well round-bottom cell culture plates. After 4 days of coculture at 37°C under 5% CO_2_, CFSE signal from T cells was acquired with BD Accuri C6 flow cytometer (BD Biosciences, NJ, USA). Data analysis was performed using the proliferation tool provided by FlowJo v10.6.2 software. Data of T cell proliferation were presented as the percentage of proliferating cells (%), precursor frequency [100% * (numbers of cells that went into division/numbers of cells at start of culture)], and proliferation index (numbers of total divisions/numbers of cells that went into division) as previously suggested (24).

### Quantitative Reverse Transcription Polymerase Chain Reaction (qRT-PCR)

Cells were lysed in TRIzol reagent (Invitrogen, MA, USA) and total RNA was extracted with 1-bromo-3-chloropropane (Cyrusbioscience, Taiwan) following its standard protocol. Complementary DNA (cDNA) was prepared using M-MLV Reverse Transcriptase (Promega, WI, USA). qRT-PCR was performed with qPCRBIO SyGreen Blue Mix (PCR Biosystems, United Kingdom). Data were acquired using the StepOnePlus Real-Time PCR System (Thermo Fisher Scientific, MA, USA). The primer sequences of genes were as follows, and glyceraldehyde-3-phosphate dehydrogenase (*GAPDH*) was used as the reference gene. *Sliced XBP-1* (*sXBP-1*), 5’-TGCTGAGTCCGCAGCAGGTG-3’ and 5’-GCTGGCAGGCTCTGGGGAAG-3’; *ATF4*, 5’-CTCCGGGACAGATTGGATGTT-3’ and 5’-GGCTGCTTATTAGTCTCCTGGAC-3’; *CHOP*, 5’- GGAAACAGAGTGGTCATTCCC -3’ and 5’- CTGCTTGAGCCGTTCATTCTC -3’; *HSPA5*, 5’-TGTTCAACCAATTATCAGCAAACTC-3’ and 5’-TTCTGCTGTATCCTCTTCACCAGT-3’; *GAPDH*, 5’- GTCTCCTCTGACTTCAACAGCG -3’ and 5’- ACCACCCTGTTGCTGTAGCCAA -3’.

### Statistical Analyses

Statistical analyses were performed using GraphPad Prism 5 software (Graphpad Software, CA, USA). In general, data were presented as mean ± standard deviation (SD). For qRT-PCR experiments, data analysis was performed following the 2^-ΔΔC^_T_ method and paired Student *t*-test. For comparison between two groups, Student *t*-test was utilized. For comparison between multiple groups, one-way analysis of variance (ANOVA) test followed by Bonferroni post test was used. Statistical significance was defined as *p*<0.05.

## Results

### THG-Induced ER Stress Responses of Oral Cancer Cells

**Figure 1:**
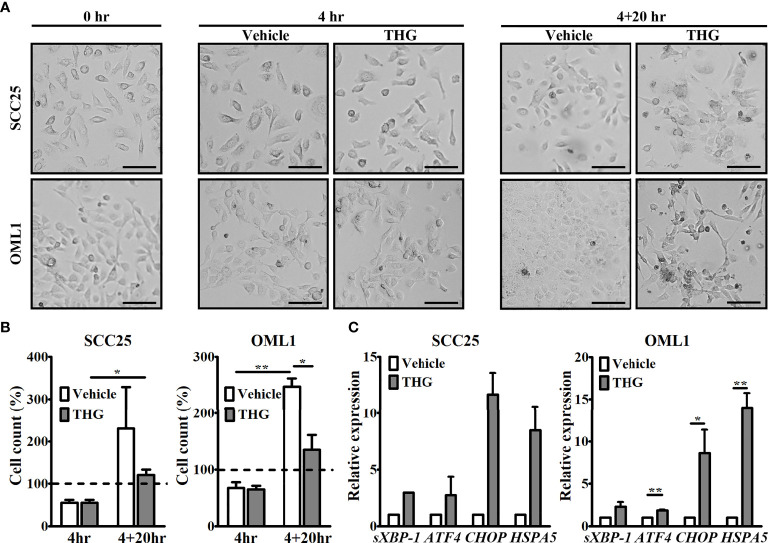
Thapsigargin (THG) induced responses of endoplasmic reticulum (ER) stress in tumor cells. **(A)** Both SCC25 and OML1 cells were treated with vehicle or 2 μM THG for 4 hours and then incubated in renewed medium for additional 20 hours. The morphology of both cell lines under light microscopy before and after treatment was shown. Scale bars represented 100 μm. **(B)** The numbers of live SCC25 and OML1 cells, determined by trypan blue stain, at 4 hours and 4 + 20 hours after treatment. Initial seeding cell number was set as 100%. **(C)** At the end of culture (4+20 hours), both SCC25 and OML1 cells were harvested to evaluate expression of ER stress-related genes, including spliced X-box binding protein 1 (*sXBP-1*), activating transcription factor 4 (*ATF4*), C/EBP homologous protein (*CHOP*), and heat shock protein family A member 5 (*HSPA5*), by quantitative reverse transcription polymerase chain reaction (qRT-PCR). The relative expression of these genes was normalized to glyceraldehyde-3-phosphate dehydrogenase (*GAPDH)* following 2^-ΔΔC^_T_ method. All data were from two biological replicates and presented as mean ± SD. *p < 0.05, **p < 0.01.

THG, a potent inhibitor of sarco/endoplasmic reticulum Ca^2+^ ATPase on ER, was used to induce ER stress of two human OSCC cell lines, SCC25 and OML1. After 4 hours of THG treatment, cells were thoroughly washed and cultured in fresh medium for additional 20 hours. Then TCM was collected at the end (4 + 20 hours). The cell morphology and cell numbers of these two cell lines were evaluated during this period. As shown in **Figure 1A**, the morphology of SCC25 cells at 4 hours or 4 + 20 hours was similar between vehicle and THG-treated groups, while THG-treated OML1 cells at 4 + 20 hours became more spindle-shaped compared with vehicle-treated groups. Moreover, the numbers of THG-treated SCC25 cells at 4 + 20 hours were higher compared with THG-treated SCC25 cells at 4 hours, indicating that proliferation of SCC25 still existed after THG treatment. In opposite, the increase in cell numbers of OML1 after THG treatment was less significant (**Figure 1B**). In addition, the ER stress responses of both cell lines after THG treatment were investigated by measuring their genetic expressions of ER stress-associated genes, *sXBP-1, ATF4*, *CHOP*, and *HSPA5*, at the end of culture (4 + 20 hours). As shown in **Figure 1C**, THG-treated OML1 cells still had higher expressions of *ATF4*, *CHOP*, and *HSPA5* compared with vehicle groups at the end of culture. On the other hand, the increase in mRNA levels *sXBP-1*, *ATF4*, *CHOP*, and *HSPA5* was less significant after THG treatment on SCC25 cells. These results indicated that THG treatment in this protocol not only induced ER stress of both cell lines, but also induced possible apoptosis especially over OML1 cells.

### ER Stressed TCM Could Promote LOX-1 Expression on Neutrophils

**Figure 2:**
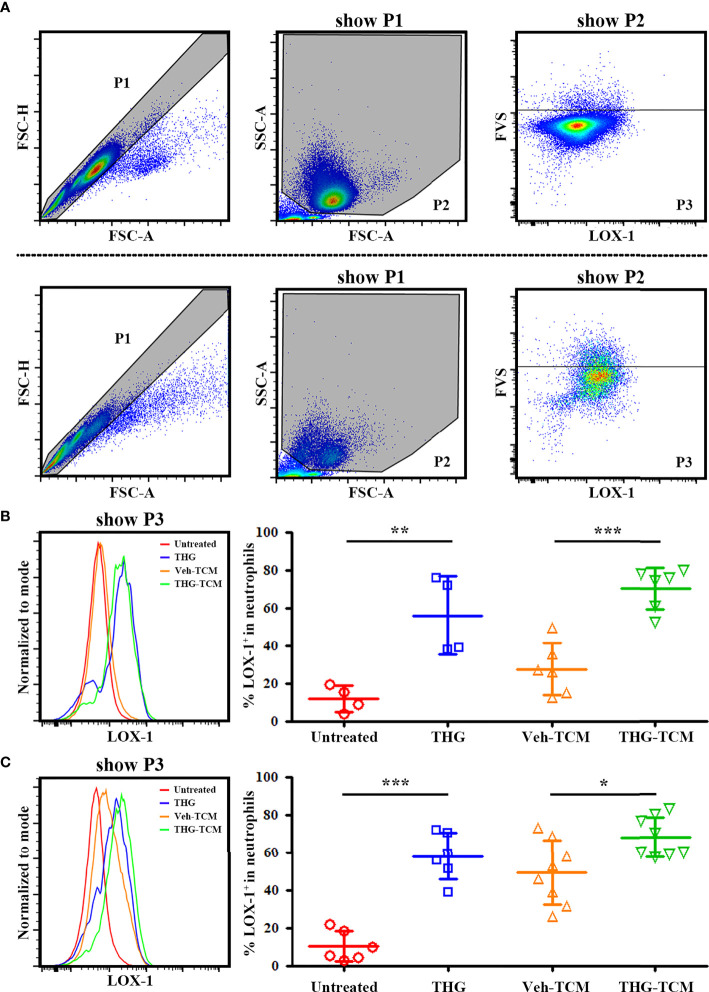
Endoplasmic reticulum (ER) stressed tumor conditioned medium (TCM) induced lectin-like oxidized low-density lipoprotein receptor-1 (LOX-1) expression on neutrophils. Both SCC25 and OML1 cells were treated with vehicle or 2 μM thapsigargin (THG) for 4 hours and then cultured in renewed medium. After 20 hours, conditioned medium was collected from vehicle-treated cells (Veh-TCM) or THG-treated cells (THG-TCM). Freshly isolated human neutrophils were then cultured with vehicle (untreated neut), 0.5 μM THG (THG-neut), Veh-TCM, or THG-TCM in the presence of 10 ng/ml GM-CSF. Untreated neut and THG-neut were served as negative or positive controls respectively. After 4 hours of incubation, LOX-1 expression on neutrophils after different treatment was evaluated by flow cytometry. **(A)** The representative gating strategy for Veh-TCM treated neutrophils (upper panel) or THG-TCM treated neutrophils (lower panel). Live neutrophils were finally gated as fixable vial stain (FVS)^-^ (P3). Then the LOX-1 expression of live neutrophils (P3) after culture in TCM from SCC25 **(B)** or OML1 **(C)** were shown. Higher percentages of LOX-1^+^ neutrophils were detected after culture in THG-TCM compared with Veh-TCM. Data were collected from 4 different **(B)** and 6 different **(C)** donors respectively and shown as mean ± SD. *p < 0.05, **p < 0.01, ***p < 0.001. FSC, forward scatter; SSC, side scatter.

To evaluate if ER stress could be transmitted from tumor cells to neutrophils and induce their expression of immunosuppressive marker LOX-1, we cultured freshly isolated human neutrophils in TCM generated from vehicle or THG treated SCC25 as well as OML1 cells for 4 hours. Nearly no carry-over THG was detected in TCM (**Supplementary Figure 1**). Neutrophils treated without or with 0.5 μM THG were served as negative or positive controls respectively. Then the expression of LOX-1 on these neutrophils was evaluated. The representative gating strategies and histograms of LOX-1 expression on neutrophils in different culture conditions were shown in **Figures 2A–C**. Indeed LOX-1 expression on neutrophils could be induced by direct THG treatment. Importantly, TCM derived from THG-treated SCC25 or OML1 cells induced higher percentages of LOX-1^+^ neutrophils as compared with TCM from vehicle-treated cells (**Figures 2B, C**). It indicated that the cell-extrinsic effect of ER stress response from tumor cells could induce LOX-1 expression on neutrophils without direct cell-cell contact.

### ER Stressed TCM Could Induce Immunosuppression of Neutrophils

**Figure 3:**
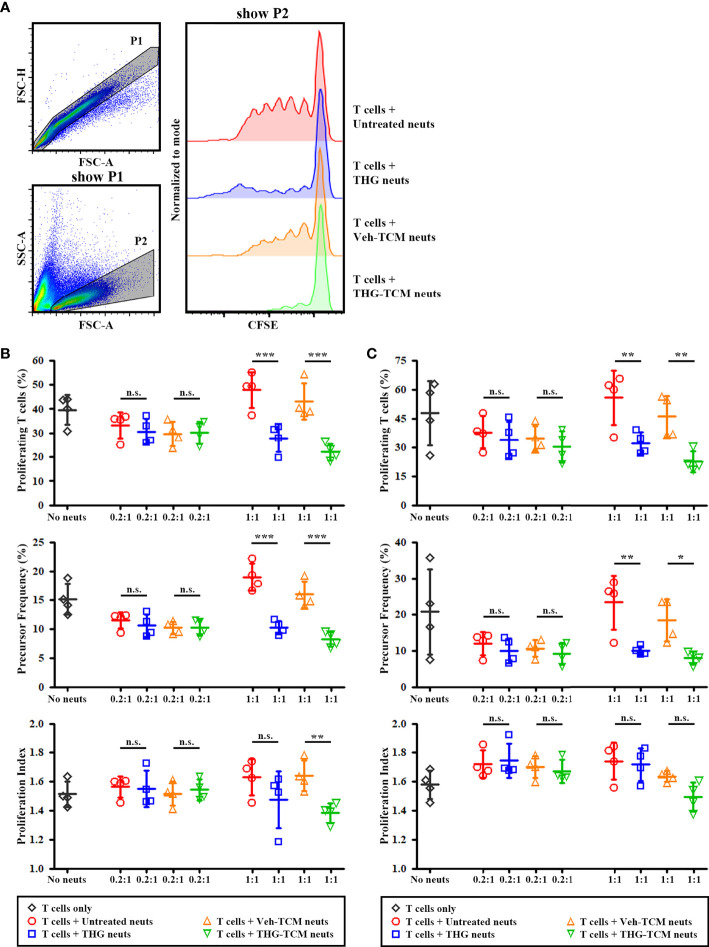
Endoplasmic reticulum (ER) stressed tumor conditioned medium (TCM) induced immunosuppressive activity of neutrophils. Human neutrophils from healthy donors were treated with vehicle (untreated neuts), thapsigargin (THG-neuts), TCM from vehicle-treated (Veh-TCM neuts) or THG-treated (THG-TCM neuts) from SCC25 or OML1 cells for 4 hours. Then these conditioned neutrophils were washed and then cultured with carboxyfluorescein succinimidyl ester (CFSE)-stained autologous T cells activated by IL-2/anti-CD3/anti-CD28 in a neutrophil/T cell ratio (N:T) of 0.2:1 or 1:1. IL-2/anti-CD3/anti-CD28 activated T cells with no neutrophils added (no neuts) were served as the positive control for T cell proliferation. At day 4 of coculture, T cell proliferation was evaluated by flow cytometry. **(A)** The representative flow cytometry plots to show CFSE staining of T cells (P2). The results of T cell proliferation after coculture with neutrophils conditioned by TCM from SCC25 **(B)** or OML1 **(C)** were presented as percentages of proliferating cells (upper panel), precursor frequency (middle panel), and proliferation index (lower panel). At N:T of 1:1, less T cells proliferated when coculture with THG-TCM neuts compared with coculture with Veh-TCM neuts. Data shown in **(B, C)** were both collected from 3 different donors and presented as mean ± SD. *p < 0.05, **p < 0.01, ***p < 0.001, n.s., non-significant. FSC, forward scatter; SSC, side scatter.

To confirm that the larger population of LOX-1^+^ neutrophils induced by ER stressed TCM were functionally more immunosuppressive, the immunosuppressive activity of neutrophils was evaluated by 4-day coculture with autologous IL-2/anti-CD3/anti-CD28 activated T cells. The representative gating strategies and CFSE histograms for T cells cocultured with different conditioned neutrophils were shown in **Figure 3A**. As expected, the percentages of proliferating T cells were lower when T cells were cultured at a ratio of 1:1 with THG-treated neutrophils compared with untreated neutrophils. This difference in T cell proliferation between untreated and THG-treated neutrophils mainly depended on the decrease in precursor frequency, not in proliferation index (**Figures 3B, C**). In addition, lower percentages of proliferating T cells were detected when T cells were cultured at a ratio of 1:1 with neutrophils cultured in TCM from THG-treated SCC25 (**Figure 3B**) or OML1 (**Figure 3C**) cells compared with neutrophils cultured in TCM from vehicle-treated cancer cells. Of note, the difference in T cell proliferation induced by TCM-conditioned neutrophils between vehicle or THG-treated SCC25 cells were due to the decrease in both precursor frequency and proliferation index (**Figure 3B**), while the difference related to vehicle or THG-treated OML1 cells was caused by the decrease in precursor frequency only (**Figure 3C**). In addition, the suppressive effect of neutrophils in T cell proliferation was not detected when the ratio of neutrophils/T cells was low (0.2:1). These results suggested that ER stressed TCM could also induce immunosuppressive activity of neutrophils.

## Discussion

In this *in vitro* study, the ER stress of OSCC has been proved to be transmitted to neutrophils, which then induces LOX-1 expression as well as immunosuppressive activity of neutrophils. This is a novel finding which further supports that the ER stress of cancer cells can manipulate nearby myeloid cells without direct cell-cell contact.

Several chemical ER stress inducers, such as tunicamycin, THG, or dithiothreitol, have been used to induce cellular ER stress *in vitro* with different mechanisms. Among these inducers, THG in the dose of 1~2μM has been shown to successfully induce ATF4, CHOP, and BiP expression of SCC25 cells (25, 26). In opposite, the data for OML1 was lacking. In this study, a short course (4 hours) of high dose (2 μM) THG followed by 20 hours of rest was chosen not only to induce ER stress but also to collect TCM of two OSCC cell lines. As a result, THG-treated SCC25 and OML1 indeed had ER stress responses with preserved cell amounts. Nevertheless, both cell lines, especially OML1, had higher genetic expression of *CHOP* after THG treatment, indicating that CHOP-mediated apoptosis may also be provoked. The potential role of cell apoptosis of THG-treated SCC25 or OML1 cells in influencing neutrophil phenotype could be further evaluated in the future.

The “transmissible” ER stress from tumor cells to especially macrophages seems to be a universal finding irrespectively of cancer cell types but the underlying mechanism has just been elucidated. The ER stress of three different murine cancer cell lines, including prostate cancer TRAMP-C1, melanoma B16F10, and Lewis lung carcinoma (LLC), could promote proinflammatory responses of macrophages through Toll-like receptor 4 (TLR4) signaling (3). In addition, another murine mammary carcinoma cell line 4T1 could also transmit ER stress to macrophages, which in turn acquired proinflammatory and proangiogenic activities (4). Moreover, the ER stress of human hepatocellular carcinoma (HCC) was also demonstrated to induce both ER stress and PD-L1 expression of macrophages *via* tumor-secreted Golgi protein 73 (GP73) (2). Although conditioned medium from human tongue squamous carcinoma cell line PCI30 failed to induce LOX-1 expression on neutrophils (8), we had successfully proved that conditioned medium from ER stressed human OSCC cell lines, SCC25 and OML1, could induce LOX-1 expression and immunosuppressive ability of neutrophils. The possible mediators for our findings may be exosomal microRNAs or GP73 produced by ER stressed OSCC. Further research is warranted to find out the underlying mechanism for transmission of ER stress between OSCC and neutrophils.

T cell proliferation assay is a common tool to evaluate the immunosuppressive ability of neutrophils *in vitro*. However, the interaction between T cells and neutrophils in this assay was highly variable depending on the parameter settings of the assay, including the origin of T cells (27) and the activation status of both T cells and neutrophils (28). In this study, a relatively simplified protocol for CFSE-based autologous T cell proliferation assay was used, but the data analysis was more delicate by calculating precursor frequency and proliferation index in addition to percentages of proliferated cells. In the end, we have successfully demonstrated that neutrophils treated by ER stressed TCM could suppress T cell proliferation, mainly due to the decrease in precursor frequency. This was supported by a recent finding that *in vitro* T cells early in the activation process are more susceptible to suppression of neutrophils than those in late activation status (28).

In conclusion, TCM prepared from ER stressed of SCC25 or OML1 cells could induce LOX-1 expression and immuno-suppressive activity of neutrophils, indicating a “transmissible” ER stress response from tumor cells to nearby neutrophils which in turn acquired immunosuppressive activity. Nevertheless, this study had some limitations. On the one hand, the underlying mechanism for transmission of ER stress from tumor cells to neutrophils was not investigated. Besides, only *in vitro* experiments were carried out in this study. Further confirmation by animal models or clinical correlations is necessary. In addition, only T cell proliferation assay was utilized to evaluate immunosuppressive activity of neutrophils. Other T cell activation assays such as production of interferon γ or expression of activation markers can be added to strengthen our findings. Despite these limitations, our findings still uncover a potential new interaction between cancer cells and neutrophils especially in OSCC.

## Data Availability Statement

The raw data supporting the conclusions of this article will be made available by the authors, without undue reservation.

## Ethics Statement

The studies involving human participants were reviewed and approved by Institution Review Board of E-Da Hospital. The patients/participants provided their written informed consent to participate in this study.

## Author Contributions

Concept and design of study: C-FW, Y-CS, P-JC, and C-CW. Acquisition of data: C-FW, T-TH, and K-HL. Statistical analysis of data: C-FW and T-TH. Drafting manuscript: C-FW and T-TH. Revising manuscript critically: C-FW, Y-CS, and C-CW. All authors contributed to the article and approved the submitted version.

## Funding

This study was supported by Ministry of Science and Technology, Taiwan (MOST 107-2314-B-650-011, 108-2314-B-650-003-MY2), E-Da Hospital, and E-Da Cancer Hospital (project EDPJ107063, EDPJ108066, EDPJ109010, EDAH108054).

## Conflict of Interest

The authors declare that the research was conducted in the absence of any commercial or financial relationships that could be construed as a potential conflict of interest.

## Publisher’s Note

All claims expressed in this article are solely those of the authors and do not necessarily represent those of their affiliated organizations, or those of the publisher, the editors and the reviewers. Any product that may be evaluated in this article, or claim that may be made by its manufacturer, is not guaranteed or endorsed by the publisher.
