# Supplementary material for: Endoplasmic Reticulum Stress of Oral Squamous Cell Carcinoma Induces Immunosuppression of Neutrophils
Source: Front Oncol. 2022 Mar 16;12:818192. doi: 10.3389/fonc.2022.818192 (PMC8966035; doi:10.3389/fonc.2022.818192)
Supplement: Supplementary file 1 [file DataSheet_1.docx]

Supplementary Material

# Supplementary Materials and Methods

**Ultra-performance liquid chromatography-tandem mass spectrometry (UPLC-MS/MS)**

A Shimazu Nexera X2 UPLC system (Shimazu, Kyoto, Japan) was applied for the qualitative and quantitative analysis of samples. Liquid chromatography was carried out utilizing a Thermo Hypersil GOLD C18 (1.9 µm, 2.1 mm × 100 mm) column (Waltham, MA, USA). The mobile phase was prepared by mixing Acetonitrile (A) and water (W, containing 0.2% formic acid), the gradient sequence was executed as follows: 0–5 min, 10–100% A; 5–10 min, 100% A. The flow rate was fixed at 0.5 mL/min, the column temperature was maintained at 40 °C. Samples were filtered through a 0.45 μm membrane filter before loading into the UPLC column. The sample injection was implemented automatically with 1 μl volume per injection. The full MS scan experiments (in the positive mode) were performed using Shimazu LCMS-8045 mass spectrometry. The dwell time was set at 100 msec and the collection was ranged from *m/z* 100 to 2000. All the acquired MS data were processed by LCMS LabSolutions software (Version 5.93, Shimazu, Kyoto, Japan).

# Supplementary Figures


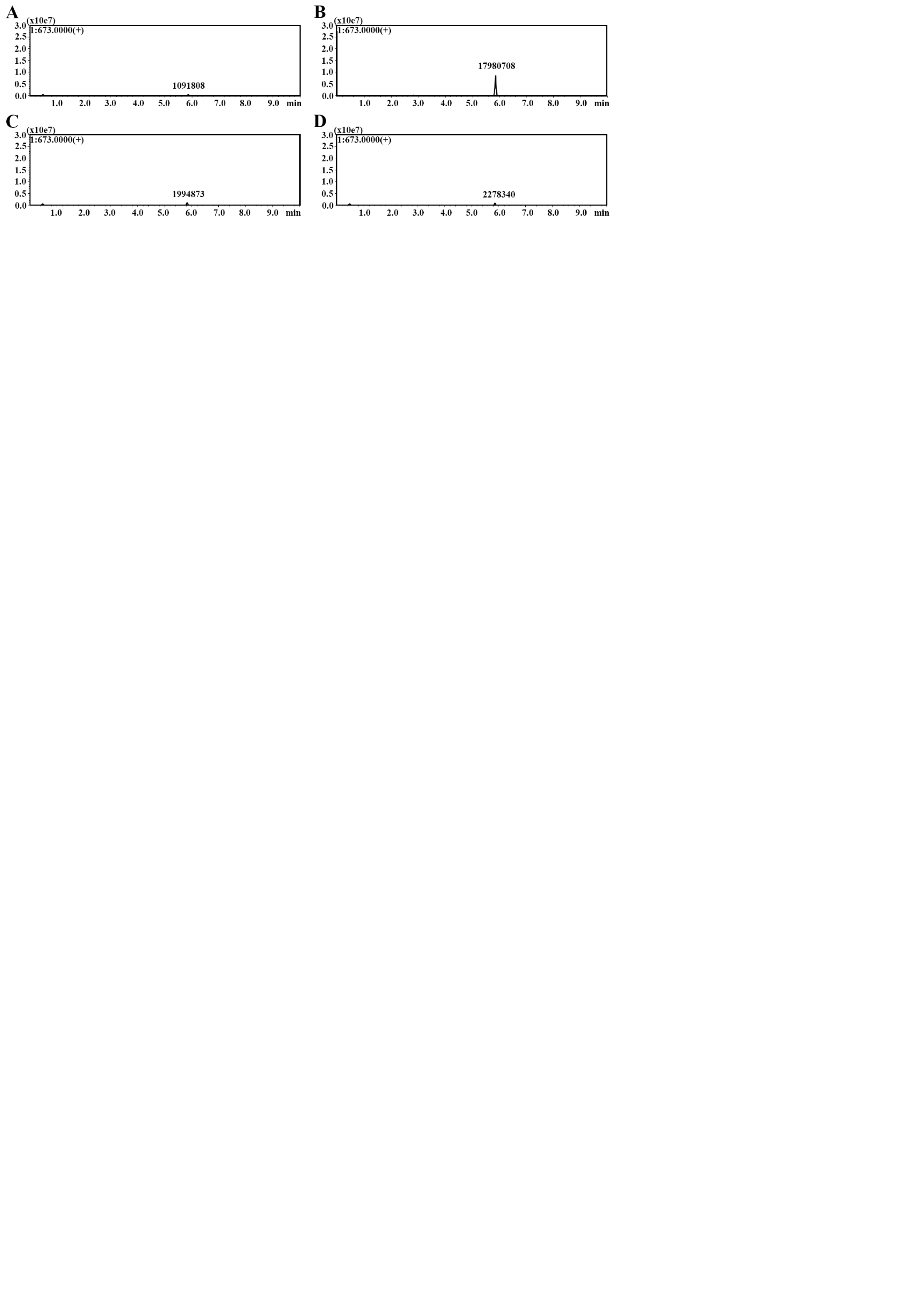


**Supplementary Figure 1 ER stressed tumor-conditioned medium (TCM) contained little carry over thapsigargin (THG).** Mass spectrometry (MS) was performed to evaluate if THG existed in (**A**) RPMI medium only, (**B**) RPMI with 1μM THG, (**C**) TCM from THG-treated SCC25, and (**D**) TCM from THG-treated OML1. The area-under-curve (AUC) values of each sample were shown respectively.
